# Reconsideration of the optimal minimum lymph node count for young colon cancer patients: a population-based study

**DOI:** 10.1186/s12885-018-4428-0

**Published:** 2018-06-01

**Authors:** Xu Guan, Yuliuming Wang, Hanqing Hu, Zhixun Zhao, Zheng Jiang, Zheng Liu, Yinggang Chen, Guiyu Wang, Xishan Wang

**Affiliations:** 1 Department of Colorectal Surgery, National Cancer Center / Cancer Hospital, Chinese Academy of Medical Sciences and Peking Union Medical College, Beijing, China; 2 Department of Colorectal Surgery, the Second Affiliated Hospital of Harbin Medical University, Harbin, China

**Keywords:** Colon cancer, Young patient, Lymph node, SEER

## Abstract

**Background:**

Currently, young colon cancer (CC) patients continue to increase and represent a heterogeneous patient group. The aim of this study was to explore the optimal minimum lymph node count after CC resection for young patients.

**Methods:**

We performed a comprehensive search of the Surveillance, Epidemiology, and End Results (SEER) database, 2360 CC patients aged from 20 to 40 were analyzed. X-tile was used to determine the optimal cut-off point of lymph node based on survival outcomes of young patients. The cancer specific survival (CSS) was estimated with Kaplan-Meier method, the Cox proportional hazards regression model was used to analyse independent prognostic factors and exact 95% confidence intervals (CIs).

**Results:**

Using X-tile analysis, 22-node measure was identified as the optimal choice for CC patients aged < 40. The 5-year CSS were 85.8% and 80.9% for patients examining ≥22 nodes and < 22 nodes. Furthermore, we identified that examining < 22 nodes was an independent adverse prognostic factor in patients aged < 40. In addition, the revised 22-node measure could examine more positive nodes than the standard 12-node measure in young patients.

**Conclusions:**

For young colon cancer patients, the lymph node examination should be differently evaluated. We suggest that 22-node measure may be more suitable for CC patients aged < 40.

**Trial registration:**

Retrospectively registered.

**Electronic supplementary material:**

The online version of this article (10.1186/s12885-018-4428-0) contains supplementary material, which is available to authorized users.

## Background

Early age at onset is often considered as a poor prognostic factor for colon cancer (CC). Young-onset CC is characterized by more advanced stages, poor tumor differentiation, mucinous carcinoma, more distal location, and even a particular profile of biomarkers [1]. Currently, CC incidence in patients younger than 50 years continue to increase, most markedly among those patients younger than 35 years by approximately 2% per year [2]. Young CC patients are affected by the disease in the prime of their life, but the life expectancy of these patients are different from older patients. In addition, there is limited knowledge about the aetiology and pathogenesis of young CC patients, especially for patients younger than 40.

The presence or absence of lymph node metastases is pivotal to the accurate staging of CC patients, thus ensuring that appropriate decisions are made regarding adjuvant therapy [3–5]. Current guidelines of the American Society of Clinical Oncology (ASCO) and the National Comprehensive Cancer Network (NCCN) advocated that a minimum of 12 lymph nodes need to be examined to establish N stage. Besides, many studies have been performed to determine the optimal number of lymph nodes that need to be examined to accurately stage CC [5–7]. However, the lymph node examination could be influenced by age, cancer site, tumor stage, and many other factors [8–10]. Therefore, large controversies still exist regarding optimal number of lymph nodes to be examined.

The clinical and biological characteristics of young CC patients are different from other age groups, thus request more attention. Some studies previously reported that young age associated with more lymph nodes to be examined [11–13], but the result remains undefined due to distinct lack of data. To better define this issue, with data from the Surveillance, Epidemiology, and End Results (SEER) database, we attempted to discuss whether the 12 lymph nodes is the optimal minimum node count for young CC patients, and further analyzed the optimal choice for lymph node examination for patients younger than 40. Secondly, we compared the superiority between the revised 22-node measure and the standard 12-node measure based on the number of positive nodes examined. Finally, we tried to identify whether this revised node measure could be considered as an independent prognostic factor for young CC patients.

## Methods

### Data source

In this retrospective study, we extracted the CC patients diagnosed between 2004 and 2013 from the SEER database. The SEER database is openly accessed, which contains detailed records on prevalence, incidence, treatment and survival outcomes of cancer cases from 18 registries in the United States. The SEER database approximately represents 28% of the US population. In addition, it also collects information regarding cancer-directed surgery, including surgical methods and extents of lymph node resection [9]. National Cancer Institute has given us permission to obtain research data file in the SEER database, and the reference number is 10249-Nov2015. The study design was approved by the Ethics Committee of the included hospitals.

### Study population

A total of 2360 CC patients aged between 20 and 40 years were collected from the SEER database. All patients were in stage I to III. Radical colectomy should be the first course of therapy for all CC patients included in this study. The surgical procedures include two modalities. 1) hemicolectomy or greater (but less than total), right or left colectomy. The hemicolectomy here is the removal of total right or left colon and a portion of transverse colon; 2) partial colectomy (less than hemicolectomy), such as enterocolectomy, ileocolectomy, cecectomy, partial resection of transverse colon and flexure and sigmoidectomy. Patients who underwent preoperative chemoradiotherapy should be excluded from this study in the consideration of the decreased number of node examined [14]. Other clinical characteristics include race, gender, tumor histology, tumor location, tumor size, tumor differentiation, AJCC TNM stage and surgical techniques. The exclusion criteria in this study were as follow: died due to other causes, whose lymph node count was unknown in the documentation and patients who received a local excision.

### Statistical analysis

The cancer specific survival (CSS) was defined as the period from the initial CC diagnosis until cancer-associated death, cancer metastasis or recurrence and the end of follow up [14]. Kaplan-Meier method was conducted to calculate the CSS, and log-rank test was used to compare the survival difference between subdivisions. Cox proportional hazards regression model was exactly performed to evaluate hazard ratios (HRs) and 95% confidence intervals (CIs) to analyse independent prognostic factors based on 5-year CSS. Differences in continuous data were analyzed using Student’s T test. Categorical data were compared by means of χ^2^ test. Statistical analyses were carried out by using the statistical software package SPSS 20.0 (IBM Corp, Armonk, NY, USA). All statistical tests were two-sided *P* values and a P value of < 0.05 was defined to be statistical significance.

The optimal cut-off point of lymph node count was determined by using X-tile plots. The X-tile demonstrated the relationship between survival outcome and different lymph node count by establishing a two-dimensional projection based on every possible cut-off point. Every possible subdivision of the populations corresponds to a χ^2^ value. The optimal cut-off point of lymph node count was determined by choosing the maximum χ^2^ value with minimum *P* value [14]. In this study, the ratio of cases in the training set vs. the validation set was 1:1. X-tile randomly generate the training set and validation set, and both of sets are normalized so that their base survival curves are similar.

## Results

### Patient characteristics

With data from SEER database, 2360 patients aged between 20 to 40 were identified, all patients were diagnosed with stage I-III CC. Baseline characteristics are shown in Table 1. The proportion of female patient was equal to male patients, while marginal larger proportions were observed in left-sided colon (51.5%), stage III (52.7%), deeper tumor invasion (55.1%) and node positive CC (52.2%). On the whole, the largest proportions were adenocarcinoma (85.7%), T3/T4 (82.1%), white (74.5%), grade II (68.7%), and hemicolectomy (61.1%).

**Table 1:** Characteristics of CC patients aged < 40 in the SEER database, 2004–2013 (*N* = 2360)

Characteristics		Number of patients	(%)
Gender	Male	1180	50.0
	Female	1180	50.0
Race	White	1758	74.5
	Black	317	13.4
	*Others	285	12.1
AJCC stage	Stage I	293	12.4
	Stage II	823	34.9
	Stage III	1244	52.7
Grade	Grade I	146	6.2
	Grade II	1622	68.7
	Grade III	517	21.9
	Grade IV	75	3.2
Histology type	Adenocarcinoma	2021	85.7
	Mucous/signet-ring cell	312	13.2
	Other types	27	1.1
Tumor location	Left-sided colon	1215	51.5
	Right-sided colon	1145	48.5
T stage	T1/T2	422	17.9
	T3/T4	1938	82.1
N stage	N0	1129	47.8
	N1/N2	1231	52.2
Tumor size (cm)	0–5	1059	44.9
	≥5	1301	55.1
Surgical procedure	Partial colectomy	917	38.9
	Hemicolectomy	1443	61.1

*Others: American Indian/AK Native, Asian/Pacific Islander

### X-tile analysis for optimal minimum node count

**Fig. 1:**
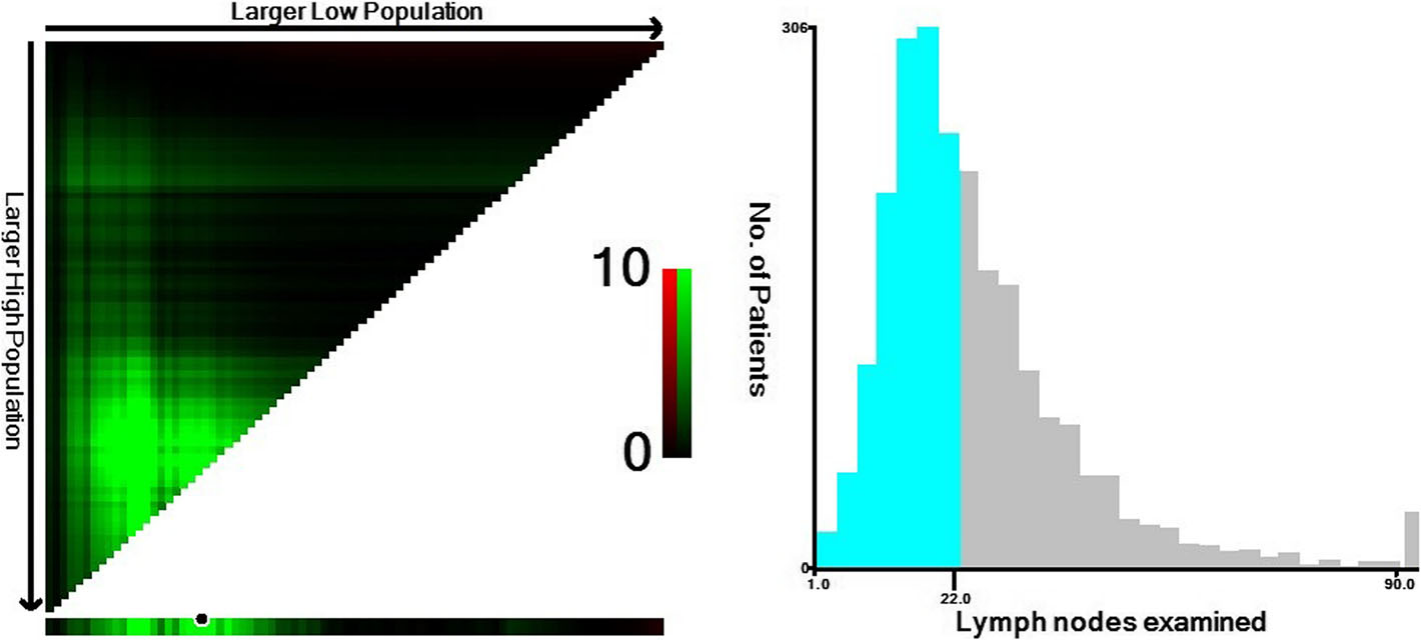
Identification of the optimal cut-off point of lymph node count for patients aged < 40. **a** The distribution of number of young patients according to lymph nodes count. Number of lymph nodes ranged from 0 to 90. **b** X-tile plots for number of lymph nodes constructed by young patients. The plots show the χ^2^ log-rank values produced, dividing them into 2 groups by the cut-off point 22. The brightest pixel represents the maximum χ^2^ log-rank value

To identify the optimal minimum node count for CC patients aged < 40, we used X-tile analysis to explore the cut-off value on the prediction of CSS based on every possible lymph node count. We found that 22 nodes was the optimal minimum node count, with the maximum Chi-square value 12.06 (Fig. 1). In further analysis, this optimal node count was used as prognostic factor.

### The effect of the 22-node measure on CSS

**Fig. 2:**
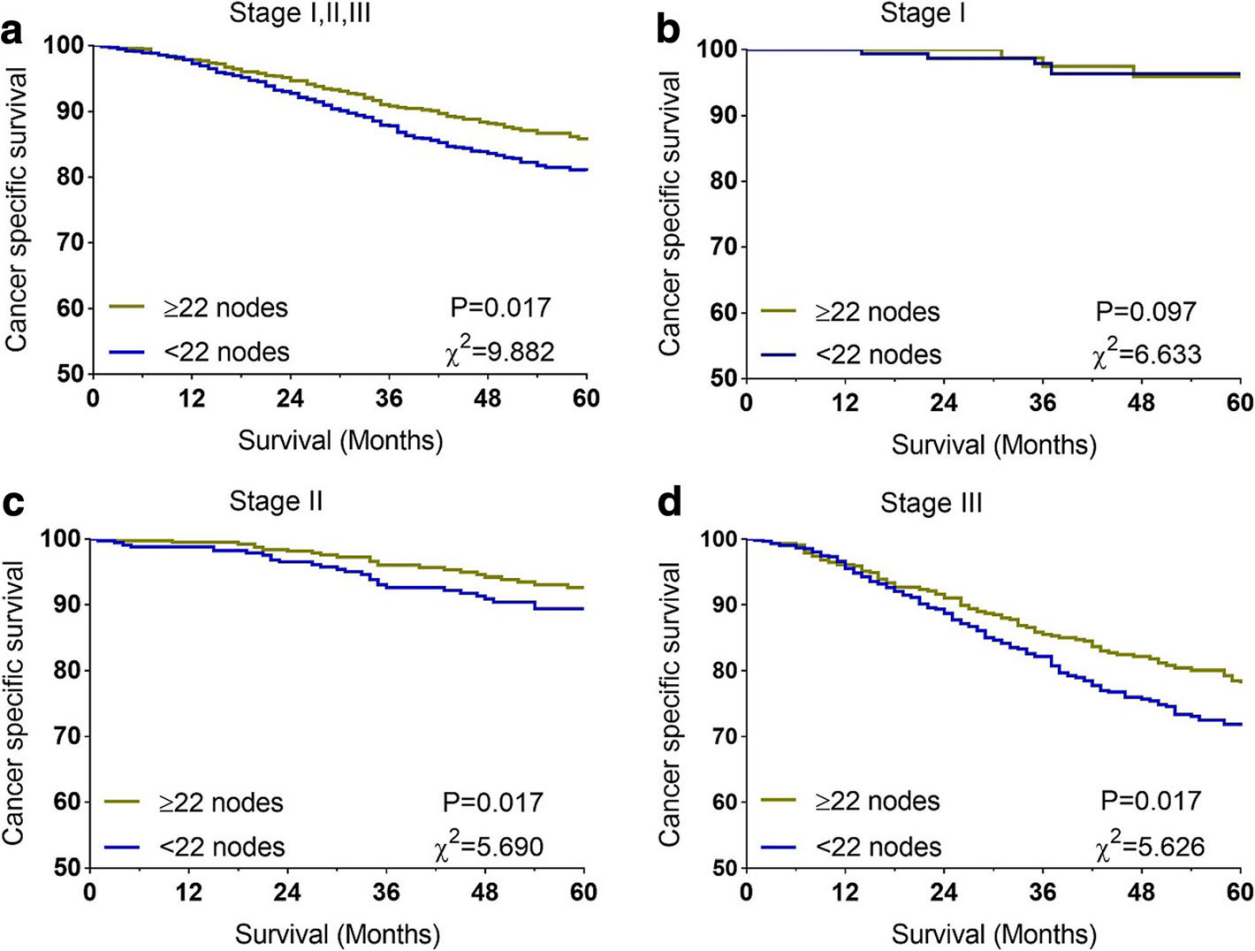
Prognostic impact of the 22-node measure on CCS for patients aged < 40. **a** 5-year CSSs in all patients between lymph node count ≥22 and < 22; (**b**) 5-year CSSs in patients with stage I between lymph node count ≥22 and < 22; (**c**) 5-year CSSs in patients with stage II between lymph node count ≥22 and < 22; (**d**) 5-year CSSs in patients with stage III between lymph node count ≥22 and < 22

For patients examined ≥22 nodes, the 5-year CSS was 85.8%, while 80.9% for patients examined < 22 nodes (*P* = 0.017) (Fig. 2a). Then, in different tumor stage, the impact of this revised node measure on CSS were identified. We found that patients in stage II and stage III could obtain more survival benefit from the 22-node measure (Fig. 2b-d).

### Univariate and multivariate regression analysis for risk factor

Univariate and multivariate regression analysis were used to explore the risk factors for survival of young CC patients. Our results identified that examined < 22 nodes was an independent prognostic factors for patients younger than 40 (Table 2). In further analysis, we identified other characteristics including female, black, deeper tumor invasion, mucous/signet-ring cell and poor tumor differentiation as independent risk factors for young CC patients. With aim to confirm the superiority of 22-node measure, we also performed the univariate and multivariate analysis based on the 12-node measure. The detailed information was shown in Additional file 1: Table S1. The results showed the HR of 22-node measure was lower than 12-node measure, which might suggest that 22-node measure could more effectively distinguish the prognosis for young CC patients.

**Table 2:** Univariate and multivariate analysis for CC patients aged < 40

Characteristics		Univariate analysis		Multivariate analysis	
		HR [95%CI]	*P*	HR [95%CI]	*P*
Nodes examined	< 22	1	0.002	1	< 0.001
	≥22	0.67 [0.54–0.84]		0.59 [0.46–0.74]	
Gender	Male	1	0.005	1	0.012
	Female	1.37 [1.10–1.70]		1.33 [1.07–1.66]	
Race	White	1	< 0.001	1	< 0.001
	Black	1.64 [1.23–2.18]		1.73 [1.29–2.31]	
	*Others	1.57 [1.16–2.13]		1.38 [1.01–1.87]	
AJCC stage	Stage I	1	< 0.001	1	< 0.001
	Stage II	2.87 [1.38–5.99]		2.82 [1.30–5.21]	
	Stage III	4.63 [3.27–6.45]		4.00 [2.89–6.05]	
T stage	T1/T2	1	< 0.001	1	0.001
	T3/T4	4.38 [2.69–7.14]		3.10 [1.59–6.07]	
N stage	N0	1	< 0.001	1	< 0.001
	N1/N2	3.67 [2.82–4.77]		3.25 [2.44–4.86]	
Tumor location	Left-sided colon	1	0.858	1	0.148
	Right-sided colon	0.98 [0.79–1.22]		1.20 [0.94–1.54]	
Histological type	Adenocarcinoma	1	< 0.001	1	0.015
	Mucous/signet-ring cell	1.76 [1.36–2.28]		1.49 [1.15–1.94]	
	Others	2.30 [0.95–5.57]		1.48 [0.60–3.64]	
Grade	Grade I/Grade II	1	< 0.001	1	< 0.001
	Grade III/Grade IV	2.22 [1.78–2.78]		1.74 [1.38–2.20]	
Surgical type	Hemicolectomy	1	0.002	1	0.002
	Segmental resection	1.45 [1.15–1.83]		1.52 [1.17–1.97]	
Tumor size (cm)	< 5	1	0.027	1	0.486
	≥5	1.29 [1.03–1.60]		1.09 [0.86–1.38]	

*Others: American Indian/AK Native, Asian/Pacific Islander

### The 22-node measure could change the migration of N stage

**Fig. 3:**
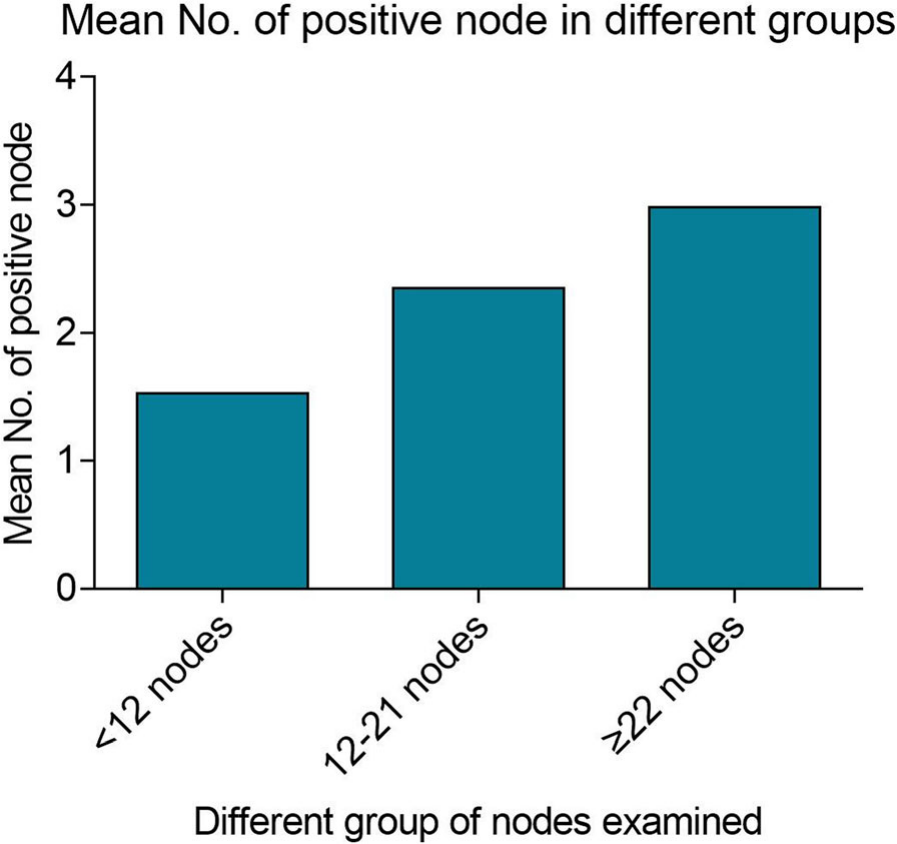
Mean number of positive node in different groups. The patients were divided into three groups according to the total number of lymph nodes examined, including < 12 nodes, 12–21 nodes and ≥ 22 nodes. The mean number of positive lymph node had a positive relationship with total number of lymph node examined, with *P* < 0.001

Finally, to evaluate the impact of 22-node measure on stage migration, we firstly divided the patients into three groups according to the total number of lymph nodes examined, including < 12 nodes, 12–21 nodes and ≥ 22 nodes. Secondly, we compared the percentage of patients in different TNM stage, N stage and the mean number of positive lymph node among three groups. The result showed that there was no difference about TNM stage migration from stage I/II to stage III among three groups, with *P* = 0.167 (Table 3). However, according different N stage, we found that the percentage of patients in stage N2 increased with more lymph nodes examined, with *P* = 0.037 (Table 3). Furthermore, the mean number of positive lymph node also had a positive relationship with total number of lymph node examined, with *P* < 0.001 (Fig. 3). Therefore, we concluded that although the 22-node measure could not change the migration of TNM stage, the N stage was obviously changed.

**Table 3:** Stage migration in different groups of lymph nodes examined

AJCC Stage		< 12 nodes (*N* = 236)	12–21 nodes (*N* = 943)	≥22 nodes (*N* = 1181)	*P*
AJCC TNM Stage	I/II	112 (47.5%)	439 (46.6%)	545 (48.9%)	0.167
	III	124 (52.5%)	504 (53.4%)	636 (51.1%)	
AJCC N Stage	N0	112 (47.5%)	439 (46.6%)	545 (48.9%)	0.037
	N1	86 (36.4%)	270 (28.6%)	326 (27.6%)	
	N2	38 (16.1%)	234 (24.8%)	310 (26.2%)

## Discussion

Although patients under the age of 40 constitute a minority of all CC patients, the incidence of CC in this age group has increased over the past decades [2, 15, 16]. Young CC patients are affected by the disease in the prime of their life, but the life expectancies of these patients are different from older patients [17]. In addition, there is limited knowledge about the aetiology and pathogenesis of young CC patients. Thus, more attention should be paid to the diagnosis and treatment for this special group of patients.

Recently, lymph node examination has been associated with accurate staging and reasonable use of adjuvant treatment [14]. For one thing, inadequate lymph node examination increases the risk of under staging and leads to unreasonable therapeutic decision which further influence the survival benefit of patients [6]. For another, the number of lymph nodes examined depends on multiple factors further influence survival [6, 11, 18–20]. Firstly, the skill of surgeon and pathologist, the extent of surgical field and the technique of pathology examination could influence the number of lymph nodes examined [6, 18–20]. These modifiable factors serve as surrogate markers for the comprehensive strength of hospital to provide better therapeutic strategy and to benefit the prognosis of patients. In addition, patients-related factors also influence the lymph node examination. Reactive lymphadenopathies and tumor characteristics, for example, represent the immune status of patients [18]. A worse immune status not only associates with more aggressive treatment and more nodes retrieved, but also associates with higher risk of recurrence and metastasis. However, limitation still exist since it’s hard to improve the survival by surgical resection for tumors with lots of lymph nodes metastasis. In general, adequate lymph node examination remains reasonable to improve survival of patients.

Several studies about identifying the minimum count of lymph nodes have being proposed to correctly classify patients into nodal negative or positive [20]. Generally, although most recommendations required at least 12 lymph nodes in CC resection, lymph node harvest remained to be highly variable. Mounting evidence have confirmed that age was considered as an independent influencing factor for lymph node examination [1, 8, 9]. Recently, young patients were noted to have more nodes retrieved in their surgical specimens than older ones [12, 13]. Furthermore, researchers found that young patients were more likely to have a nodal yield of ≥12 nodes [11, 21]. Compare with older patients, young-onset CC is characterized by more advanced tumor stage, more aggressive histopathologic features, higher positive rate and more extended resections, which might be the potential reasons for more nodes retrieved [13]. Interestingly, these results were identical to our previous study [14]. Our previous study had shown that the median number of lymph node count was decreased with increasing age, which were 25.5, 20.2, 17.8 and 16.9 for patients aged 20–39, 40–59, 60–79, and ≥ 80, respectively (*P* < 0.001) [14]. The decreased node count may result from a stronger immunological response to malignant tumor in young patients and more extended resections compare with older patients [14]. Although the potential reasons remain undefined, these results remind us that retrieve at least 12 lymph nodes was not enough for young CC patients and 12-node measure need to be revised.

In this study, we explored the optimal cut-off value for CC patients younger than 40 based on the prediction of CSS. Firstly, we identified 22-node measure as the optimal choice for patients aged < 40. According to survival outcome, patients in stage II and stage III could obtain more survival benefit by using 22-node measure. In addition, we identified examining < 22 nodes as an independent adverse prognostic factor for young patients. Compared with the 12-node measure, 22-node measure could more effectively distinguish the prognosis of young CC patients. Finally, 22-node measure changed the migration of N stage. Accordingly, we considered that 22-node measure might be more suitable for young CC patients aged < 40. Besides, we also evaluated the time-dependent changes in lymph node yield from 2004 to 2013 to further evaluate the potential impact of improvements of surgical and pathological techniques on lymph node examination. A significant difference in lymph node yield was observed over time (Additional file 2: Figure S1).

Currently, the SEER is regarded as one of the best population-based databases, and it maintains stringent quality control measures to prevent coding errors. However, there are still defects in this cohort study. Firstly, in univariate and multivariate analysis, the type and distribution of surgery could also be influencing factors of lymph node examination since the minimal invasive operations and open laparotomies could influence the surgical approach and exploration scope. Secondly, detailed information with regard to chemotherapy and radiotherapy were not provided in SEER database, which could also influence the prognosis of CC patients. Finally, we could not avoid the selection biases since this study belonged to retrospective cohort study. Despite these limitations, SEER remains a valuable resource to analyze trends and patterns in patient characteristics, tumor features, cancer treatments as well as survival outcomes.

## Conclusion

In conclusion, patients under the age of 40 constitute a minority of all CC patients, but the incidence of CC in this age group has increased over the past decades. Compared with older patients, young patients often retrieved more than 12 nodes. Based on our results, we suggested that patients younger than 40 should examined no less than 22 nodes instead of 12-node measure. However, whether this revised 22-node measure could impact the adjuvant treatment decision-making for young patients, the current study could not provide a satisfactory answer, and more prospective studies focused on this group of patients should be designed in the future.

## Additional files

Additional file 1: **Table S1.** Univariate and multivariate analysis for CC patients aged < 40. (DOCX 18 kb)

Additional file 2: **Figure S1.** The time-dependent changes in lymph node yield from 2004 to 2013. (JPG 150 kb)

## Abbreviations

AJCC: American Joint Committee on Cancer
ASCO: American Society of Clinical Oncology
CC: Colon cancer
CI: Confidence interval
CSS: Cancer specific survival
HR: Hazard ratio
NCCN: National Comprehensive Cancer Network
SEER: Surveillance, Epidemiology, and End Results.
