# Supplementary material for: Reconsideration of the optimal minimum lymph node count for young colon cancer patients: a population-based study
Source: BMC Cancer. 2018 Jun 1;18:623. doi: 10.1186/s12885-018-4428-0 (PMC5984774; doi:10.1186/s12885-018-4428-0)
Supplement: Supplementary file 1 — Table S1. Univariate and multivariate analysis for CC patients aged < 40. (DOCX 18 kb) [file 12885_2018_4428_MOESM1_ESM.docx]

**Supplementary Table 1** Univariate and multivariate analysis for CC patients aged <40.

| Characteristics | | Univariate analysis | | Multivariate analysis | |
| --- | --- | --- | --- | --- | --- |
|  |  | HR [95%CI] | P | HR [95%CI] | P |
| Nodes examined | <12 | 1 | 0.010 | 1 | <0.001 |
|  | ≥12 | 0.68 [0.51-0.91] |  | 0.57 [0.43-0.78] |  |
| Gender | Male | 1 | 0.005 | 1 | 0.010 |
|  | Female | 1.37 [1.10-1.70] |  | 0.75 [0.60-0.93] |  |
| Race | White | 1 | <0.001 | 1 | <0.001 |
|  | Black | 1.64 [1.23-2.18] |  | 1.82 [1.36-2.43] |  |
|  | Others | 1.57 [1.16-2.13] |  | 1.40 [1.03-1.90] |  |
| AJCC stage | Stage Ⅰ | 1 | <0.001 | 1 | 0.890 |
|  | Stage Ⅱ | 2.87 [1.38-5.99] |  | 2.70 [1.15-5.14] |  |
|  | Stage Ⅲ | 4.63 [3.27-6.45] |  | 4.11 [2.98-6.21] |  |
| T stage | T1/T2 | 1 | <0.001 | 1 | 0.001 |
|  | T3/T4 | 4.38 [2.69-7.14] |  | 3.12 [1.59-6.10] |  |
| N stage | N0 | 1 | <0.001 | 1 | 0.782 |
|  | N1/N2 | 3.67 [2.82-4.77] |  | 3.31 [2.48-4.91] |  |
| Tumor location | Left-sided colon | 1 | 0.858 | 1 | 0.126 |
|  | Right-sided colon | 0.98 [0.79-1.22] |  | 0.83 [0.65-1.06] |  |
| Histological type | Adenocarcinoma | 1 | <0.001 | 1 | 0.016 |
|  | Mucous/signet-ring cell | 1.74 [1.33-2.28] |  | 1.46 [1.11-1.92] |  |
|  | Others | 2.30 [0.95-5.57] |  | 1.67 [0.74-3.81] |  |
| Grade | Grade Ⅰ/Grade Ⅱ | 1 | <0.001 | 1 | <0.001 |
|  | Grade Ⅲ/Grade Ⅳ | 2.22 [1.78-2.78] |  | 1.73 [1.37-2.19] |  |
| Surgical type | Hemicolectomy | 1 | 0.002 | 1 | 0.004 |
|  | Segmental resection | 1.45 [1.15-1.83] |  | 0.68 [0.53-0.89] |  |
| Tumor size (cm) | <5 | 1 | 0.027 | 1 | 0.650 |
|  | ≥5 | 1.29 [1.03-1.60] |  | 1.06 [0.83-1.34] |  |

*Others: American Indian/AK Native, Asian/Pacific Islander.
