# Supplementary figures and images for: Reconsideration of the optimal minimum lymph node count for young colon cancer patients: a population-based study
Source: BMC Cancer. 2018 Jun 1;18:623. doi: 10.1186/s12885-018-4428-0 (PMC5984774; doi:10.1186/s12885-018-4428-0)

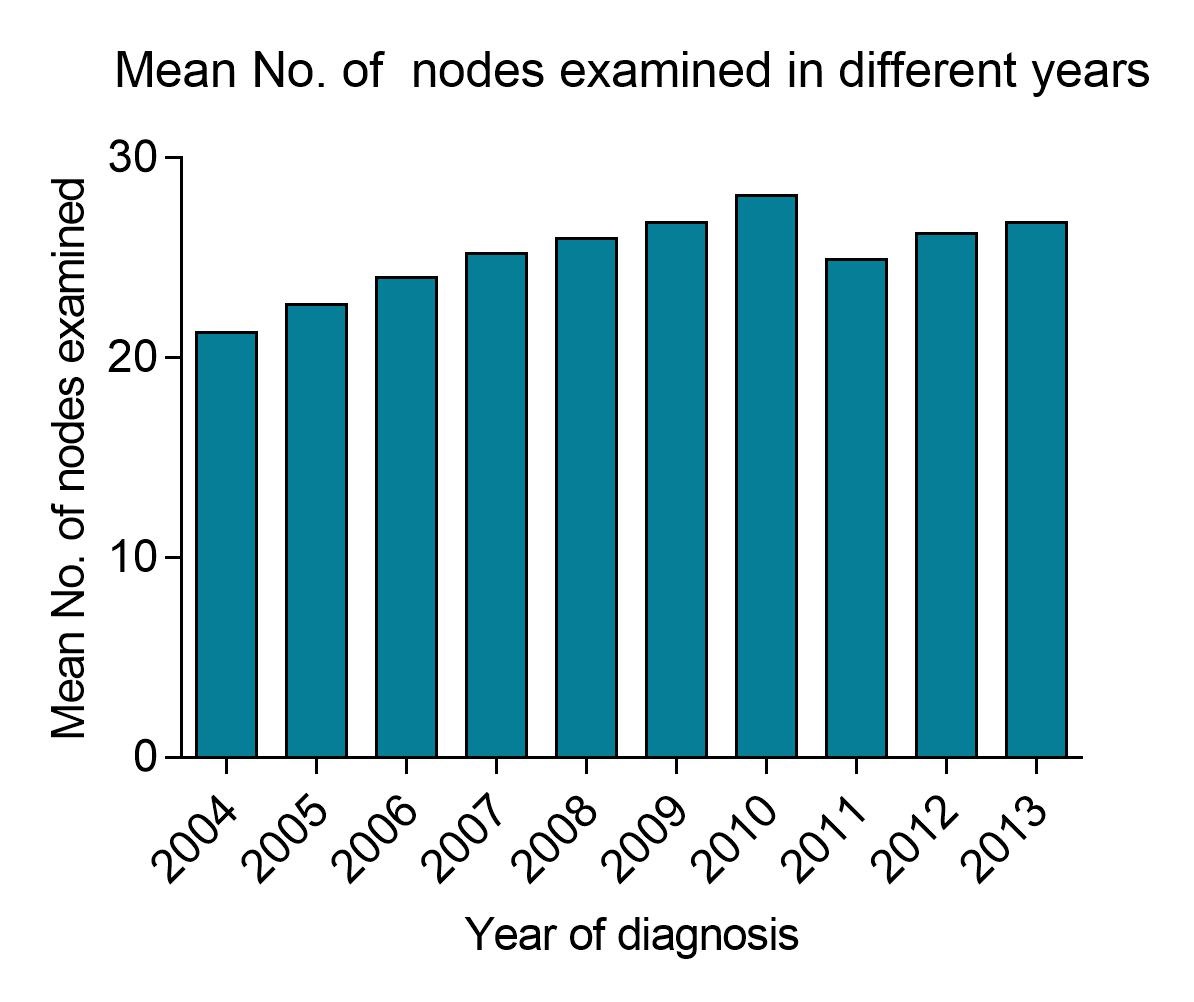

Supplement: Supplementary file 2 — Figure S1. The time-dependent changes in lymph node yield from 2004 to 2013. (JPG 150 kb) [file 12885_2018_4428_MOESM2_ESM.jpg]
